# Editorial: Exploring genetic and molecular pathways in plant reproduction for enhanced crop traits

**DOI:** 10.3389/fpls.2025.1745072

**Published:** 2025-12-03

**Authors:** Sailaja Bhogireddy, Akshaya Kumar Biswal, Emidio Albertini

**Affiliations:** 1 Department of Horticulture, The University of Georgia, Tifton, GA, United States; 2 Institute of Plant Breeding, Genetics and Genomics, University of Georgia, Tifton, GA, United States; 3 Department of Agricultural, Food and Environmental Sciences, University of Perugia, Perugia, Italy

**Keywords:** plant reproduction, multi-omics, molecular pathways, genome editing, apomictic

Plant reproduction plays a vital role in crop improvement, directly influencing both the quality and quantity of seed production. Various factors govern plant reproductive processes, which ultimately determine successful seed set and yield. Biotic and abiotic stresses affect both sexual and asexual reproduction and thus impair seed set and productivity (Ma et al., 2020; Begcy et al., 2024). Key reproductive events, such as gametogenesis, fertilization, embryogenesis, and seed maturation, are tightly regulated by gene networks, epigenetic mechanisms, hormonal signaling, and environmental factors (Zakharova et al., 2022; Peer et al., 2025). Dissecting these pathways at the molecular level provides critical insight into how fertility, hybrid vigor and apomixis are regulated. An in-depth understanding of genetic and molecular pathways underlying reproductive development is crucial to address these challenges and opens the possibilities to improve crop traits (RECROP COST, 2025).

Recent advances in plant biotechnology and multi-omics studies have revolutionized our ability to probe these complex processes. Genomic and functional-genomics approaches help reveal gene networks and structural variation underlying reproductive traits. Gene editing, including base editing and prime editing, has emerged as a powerful toolkit to precisely alter reproductive genes (Toda et al., 2023). Combined with transcriptomics, metabolomics, epigenomics, and systems biology, these technologies are now enabling a holistic view of plant reproduction, bridging the gap between basic molecular insights and translational breeding strategies. This Research Topic, ‘*Exploring genetic and molecular pathways in plant reproduction for enhanced crop traits*’ seeks to bring together these emerging insights and showcase works that push the frontier of how we can engineer durable reproduction traits into crops.

Apomixis, a form of asexual reproduction that enables the formation of clonal seeds without fertilization, has high significance for its potential applications in agriculture. In this special edition, Pasten et al. provided new developmental insights into the coexistence of sexual and apomictic pathways. The authors investigated the reproductive development of *Eragrostis curvula* (Schrad.) Nees, a perennial grass exhibiting both sexual and diplosporous apomictic cytotypes. Using confocal laser microscopy, they provided the first detailed comparative description of female and male gametophyte development in three genotypes, fully apomictic- Tanganyika, facultative apomictic- Don Walter, and sexual- OTA-S. Their analysis revealed distinct morphological traits, including larger ovules in the sexual genotype following meiosis. In addition, expression profiling of SQUAMOSA PROMOTER BINDING PROTEIN-LIKE 7 (SPL7) showed its overexpression in the sexual genotype, suggesting a potential regulatory role in reproductive differentiation. Their work reveals the cellular and molecular signatures that distinguish these two modes, offering a roadmap to exploit apomixis for fixing hybrid vigor in grasses.

Additionally, Bao et al. performed an integrated transcriptomic and metabolomic analysis to unravel the molecular basis of sexual and apomictic embryo formation in *Juglans regia* (Persian walnut), a crop of high economic and nutritional value. The study identified 321 differentially expressed genes (DEGs) and 19 differentially accumulated metabolites (DAMs) between apomictic and sexual embryos. These were primarily enriched in pathways related to secondary metabolism, hormone signaling, and tryptophan metabolism. Key metabolites, such as tryptamine, jasmonic acid (JA), and JA-isoleucine, and associated genes including *BAK1*, *trpB*, *AOC3*, *TDC*, *ZEP*, and *JAZ* were found to play regulatory roles in apomictic embryo development. This work provides the first multi-omics framework for understanding apomixis in walnut, revealing candidate genes and metabolites that may govern asexual reproduction in woody perennials. This dual-omics perspective enriches our understanding of perennial species and demonstrates how molecular dissection can guide the deployment of apomixis in clonally propagated crops.

Farinati et al. presented a case study in tomato, where targeted gene editing was used to induce male sterility (MS) using CRISPR/Cas9-mediated gene editing in tomato (*Solanum lycopersicum*). This study provides a valuable model for hybrid seed production in horticultural crops. Male sterility, defined as the inability to produce functional pollen or fertile sperm cells, is a key trait for exploiting heterosis in F_1_ hybrids. The authors targeted the *MYB80* gene, a critical regulator of pollen development, using a protoplast-based, DNA-free gene-editing approach that directly delivered Cas9–sgRNA ribonucleoprotein complexes. Their results demonstrated site-specific mutagenesis and validated *MYB80* as a promising candidate for engineering MS. This study not only advances precision breeding technologies in tomato but also establishes a reproducible platform for developing hybrid systems in other horticultural crops.

Rice, a global staple and model plant, offers unique opportunities to link fundamental reproductive biology with crop performance. Chen et al. characterized the *OsLAP3*/*OsSTRL2* gene, encoding a *strictosidine synthase*-like protein, as a critical regulator of anther cuticle formation and pollen exine patterning in rice. A male-sterile mutant (*lap3*) displayed normal vegetative growth but complete male sterility due to delayed tapetal programmed cell death (PCD) and disrupted lipid metabolism during anther development. Through map-based cloning, CRISPR/Cas9 mutagenesis, and complementation, the authors confirmed that a two-nucleotide deletion in *OsLAP3* causes this phenotype. Functional assays revealed that *OsLAP3* localizes to the endoplasmic reticulum and is essential for the biosynthesis and transport of lipid polymers, including waxes and cutin. Comparative analyses indicated that *OsLAP3* is homologous to *ZmMS45*, a core gene in maize Seed Production Technology (SPT) and its loss leads to downregulation of anther/pollen development genes, alongside pronounced reductions in wax and cutin accumulation. Lipid profiling and expression analyses together highlight *OsLAP3* as a nexus for fatty-acid–derived polymer biosynthesis, needed for fertility. These findings clarify molecular control of lipid-mediated pollen wall formation and position *OsLAP3* as a promising target for genic male sterility and hybrid rice breeding applications.

This Research Topic brings together studies spanning grasses, tree crops, and major cereals, highlighting both conserved mechanisms and species-specific innovations. Collectively, these contributions underscore the promise of integrative molecular approaches in apomixis, hybrid seed production, and fertility regulation. The articles in this Research Topic collectively enrich our understanding of plant reproduction, presenting both conceptual advances and translational opportunities. They exemplify how a multidisciplinary approach, bridging genetics, molecular biology, and biotechnology, can transform plant breeding. We anticipate that these contributions will inspire further research aimed at unlocking the full potential of reproductive pathways for sustainable agriculture.
